# University without Borders

**DOI:** 10.1007/s12124-022-09714-3

**Published:** 2022-07-02

**Authors:** Luca Tateo, Giuseppina Marsico, He Min, Maria Virginia Machado Dazzani

**Affiliations:** 1 Department of Special Needs Education, University of Oslo, Sem Sælands vei 7, Helga Engs hus, 0371, Oslo, Norway; 2 University of Salerno, Salerno, Italy; 3 East China Normal University, Shanghai, China; 4 Federal University of Bahia, Salvador, Brazil

**Keywords:** Student international mobility, Decolonizing higher education, Cultural psychology, Methodology, Research-tandem dissertation

## Abstract

The paper discusses the problem of master theses’ production in psychology from a decolonial perspective. It presents a critique to the reproductive and monological model of knowledge currently promoted in higher education. Then, it proposes an alternative pedagogic model of research-tandem. The research-tandem is an example of an innovative way of understanding a university without borders, as developed within the international network of excellence “IBEF- Ideas for the Basic Education of the Future”. Higher education must be detached from national-based curricula, and become a nomadic and collaborative across-cultural knowledge building endeavor. Current higher education aims to be national in its curricula but global in its marketability. In cultural psychology’s perspective, higher education of the future shall be regarded as global in its vision yet local in its solutions. Future students must have the opportunity to build new knowledge by experiencing and sharing diversity rather than complying with standardized and monological trajectories.

## Introduction

Szulevics, Lund and Lund (2021) ask an interesting question: why do the Danish master students in psychology choose more often to use a qualitative approach when they have to write their master thesis? This appears to be in contrast with the fact that quantitative and experimental approaches are currently mainstream in psychological sciences. To answer the question, Szulevics, Lund and Lund (2021) describe the Danish curriculum in psychology and the rules for writing a master thesis. Then, they present the results of a mixed-method study analysing the problem’s statements of 4,400 master theses contained in the official databases of the Danish universities. According to Szulevics, Lund and Lund (2021), the results are somehow surprising: a large part of the master thesis use a qualitative approach (30%) or develop a theoretical approach (14%). These results seem to confute the current mainstream view of psychology as mainly quantitative and experimental empirical science. The results show that Danish psychology students have a marked preference for qualitative approaches over quantitative ones. Szulevics, Lund and Lund (2021) try to explain this seeming paradox by considering the long-lasting debate between quantity and quality in psychology and the structural reasons that lead Danish students to prefer qualitative methods.

## Conservative revolutionaries

Universities are institutions devoted to developing innovation that are also very conservative (McLennan, 2008; Valsiner et al., 2018). Universities are also constantly challenged to meet the call of neoliberal demands, which often turns them into institutions that prioritize market demand by training a qualified workforce. Consequently, higher education institution tend to reduce their innovation investments on humanistic and social education (Battaly, 2014; Dazzani et al., 2020). The rhetoric of innovation and quality hides resistance to change, formalism and conformism (Tateo, 2018). If Szulevics, Lund and Lund (2021) had considered the everyday practices of Danish psychology programs would have probably realized that the process of writing a master thesis is framed by a number of formal constraints, rules and templates that kill creativity and lead students to stay in their comfort zones by repeating what has been already done before.

The academic conservatism is also expressed in rigid disciplinary boundaries, ways of evaluating and grading that favour reproductive writing rather than innovation. Students worry about grades and performances, which are the most important element of evaluation. If there is an institutional pressure to conform and reproduce, why should students take the risk of trying something unusual? This results in what Matthiesen & Wegener (2019) call well-written boring theses. As any educational institution, universities send ambivalent messages to students (Gomes et al., 2018; Tateo, 2018): “be creative but do not write outside the lines”; “work in group but be assessed individually”; “choose your own problem statement but don’t go out of your discipline’s borders”; “be critical but don’t ask stupid questions; “be international but study only in your national language”; etc. This is of course not a peculiarity of the Danish academic context studied by Szulevics, Lund and Lund (2021). Universities are also institutions that share some common values and practices while being very nationalistic. Two cultures overlap in higher education. On the one hand, the shared academic culture, sometimes dating back centuries with its own values, rituals, typical characters, power relations, architectures, oppression and conservatism but also moments of sudden breakthrough. The ambivalent nature of the mainstream academic culture of the Global North was nicely depicted in the popular series “The Chair” (Steinberg, 2021), in which every scholar could recognize a familiar character. Silva Guimarães (2022) talked about academic rites and myths that build an apparent state of peace, mediating between an institution that tends to perpetuate itself through crystallized practices and a continuous turnover of students’ cohorts that need to learn the academic code of communication. The dialectic between stability and change, inclusion and exclusion may be more relevant to understand the academic life rather than old-fashioned binaries such as quantitative/qualitative.

On the other hand, Humboldtian universities were imbued with national identity, and the post-Humboldtian neo-liberal competitive model is further promoting the interpretation of universities’ ranking in nationalistic sense (Ash, 2006).

Moreover, Szulevics, Lund and Lund (2021) do not consider another important aspect of the actual academic practice in Denmark. Since the higher education reform in 2015, which introduced the new public management model, the socialdemocratic Scandinavian and the neo-liberal tradition, the rules of thesis supervision were built in such a way to inhibit a form of craftsmanship and affective mentoring supposedly to provide a fair and equal treatment to all the students. For instance, if one reads the master thesis writing and supervision rules at the Danish university of Aalborg (AAU Studienævnet, 2021). It is available only in Danish, of course, yet one can see how the master thesis writing is strictly regulated, including for instance the fact that the supervisor must approve the list of references the student wants to use. The student handbook provides guidelines for the content, length, table of content of the master thesis (AAU Studienævnet, 2021). Sticking to those formal rules is subject to the censor evaluation and thus determines the final grade of the thesis. If the student does not comply, their work will be considered not well-written. Now, imagine a student who wants to adopt an innovative qualitative research methodology such as art-based approaches. The format of the master thesis will never fit into the existing framework and may be under evaluated. So, it is not surprising that, as Szulevics, Lund and Lund (2021) note, qualitative methods in MA theses are often limited to few semi-structured interviews that are well-established and acceptable to any academic tradition. This leads to a reproductive and conservative attitude towards the methods of data construction and analysis. Students are not urged to reflect on the means by which they produce their data, let alone the theoretical assumptions that support them.

The choice of the master thesis supervisor is not completely up to the student and it is formally the department that assigns a supervisor to the student, on the basis of a generic match with the thesis topic. There are few cases in which student express a preference for a specific supervisor, a professor that maybe had a particular charisma or is working on a project of interest.

Moreover, in the current regulation, all the students have the right to the same amount of supervision hours (in Scandinavia is average 10 h). It is considered unfair to ask for supervision exceeding the amount. Yet, any teacher knows that every student need is different. Some students require more guidance and emotional support; some work in a more independent way; some need a dialogue to elaborate ideas; etc. A rigid organization of supervision’s hours is required only by accounting needs but not by pedagogical needs. Such a rigid organization implicitly limits the reciprocal demands between students and their supervisors. On the one hand, time is a border pole that delimits both the boundaries of the supervisor’s work and the limits of the student’s access to their supervisor.

The academic rules frame the pedagogical relationship between supervisor and student as an impersonal and distant one in order to provide a fair and equal treatment. Actually, they defuse the affective dimension of pedagogical mentoring and turn the master thesis into the umpteenth instrumental action towards a qualification for the labour market. In such an instrumental and an affective framework, it is not surprising that the master students choose to follow the familiar reproductive path of standardized theses. It is fully compliant with the instrumental logic of productivity: obtaining the maximum outcome with the minimum effort in the shortest time. Besides the issues in the research design – as for instance the fact that in the 48% of the theses analysed it is not possible to determine the methodological approach, Szulevics, Lund and Lund (2021) focus only on the outcome without considering the whole ecosystem a master thesis is part of and the complex processes involved in this intellectual production. As all the conservative revolutionaries inspired by good will, they do not take their premises to the radical consequences.

## University without borders

The radical question is what is the real function of master thesis? Do students really need this task in its present form? Is it still an intellectual endeavour that shows the student’s intellectual maturity, their capability of independent and critical thinking and their research skills? Alternatively, is it the final symbolic act marking the end of the liminal process of higher education?

The most part of the current symbolic practices in academia worldwide are meant to set up disciplinary, temporal, ethical and identity borders (Marsico, 2018; Tateo & Marsico, 2021).

“[U]niversities today are large systems of authoritative control, standardization, gradation, accountancy, classification, credits and penalties. We need to decolonize the systems of access and management insofar as they have turned higher education into a marketable product, rated, bought and sold by standard units, measured, counted and reduced to staple equivalence by impersonal, mechanical tests and therefore readily subject to statistical consistency, with numerical standards and units.” (Mbembe, 2016, p. 30)

The current system of accountability and rewarding in Western universities is based on the fact that students should graduate on schedule and with good grades in function of their employability. Any other purpose is penalizing: no time for roaming around subjects; following intellectual curiosity; building a well-centred personality structure; travelling; etc. Thus, the question whether psychology students choose qualitative or quantitative methods for the master thesis sounds like the last of the problems. Achille Mbembe’s question (2016) is crucial: what is higher education aiming at? Is it encouraging “students to develop their own intellectual and moral lives as independent individuals”; redistributing “as equally as possible a capacity of a special type – the capacity to make disciplined inquiries into those things we need to know, but do not know yet” and “the capacity to make systematic forays beyond our current knowledge horizons”? (Mbembe, 2016, p. 30) Alternatively, does it “prevent the realization of this goal”? (*ibidem*)

The elephant in the room that Szulevics, Lund and Lund (2021) do not address is that the current system is producing undergraduate students in psychology who go out in the labour market without having the time to mature intellectually and emotionally. They cross the university, grab some knowledge in function of employability and plan to come back every now and then to update their “sandbox”. What is the role of the master thesis in this tragic path? Probably none except to symbolically sanction that the student “fits”.

Echoing Mbembe’s (2016) words: “We have to decolonize this because it is deterring students and teachers from a free pursuit of knowledge. It is substituting this goal of free pursuit of knowledge for another, the pursuit of credits. It is replacing scientific capacity and addiction to study and inquiry by salesman-like proficiency.” (p. 30).

So, let’s try to draw some “radical” consequences, and discuss how an alternative model could look like.

## Who can create new knowledge?

Knowledge grows in dialogue and across borders (Silva-Filho & Dazzani, 2016; Tateo & Marsico, 2021). Overabundant restrictions and borders of any sort – disciplinary, temporal, economic, etc. - are placed on students’ intellectual curiosity and free exploration by neo-liberal academic institutions. The sake of the accountability and the standardization of diplomas is killing the intellectual effort to produce new system of thoughts and promoting conformism and monological discourse. A discourse that often echoes in the universities’ meeting rooms and corridors is that students are not ready or qualified enough to take certain initiatives, such as publishing or using complex methods, not to mention developing their own new ideas or theories. They should reproduce rather than producing yet they are considered ready to go out in the job market as psychologists once their MA thesis is approved. Higher education should help humans in developing multiple codes to make sense of experience. An academic degree does not automatically make a student better or more mature than any other fellow. It can barely provide the means to overcome a standardized and common sense modality of understanding reality. The tendency is rather to turn higher education into a military, religious or factory-like system educational setting, where the adherence to certain credo, standard or set of rules is more valued than any exploration of new possible worlds (Marsico, 2015; Szulevicz et al., 2016; Mbembe, 2016).

## How to cultivate intuition and diversity: the example of the research-tandem

Is such a tendency irreversible? Is it the production of MA thesis barely becoming a matter of bounded choices, in which the students is required to reproduce a limited range of theories and methods, no matter if qualitative or quantitative? Of course, a scientific attitude does not imply the mere *pars destruens* of critical thinking. It also requires the *pars construens* of experimenting innovative and effective solutions to decolonize higher education and promote diversity and polyphonic thinking.

Different solutions can be implemented to overcome the academic borders and the domination of Global North episteme in psychology. One example is the method of *research-tandem* international students’ mobility implemented for MA theses (Xu & Marsico, 2020). The research-tandem is based on the assumption that diversity matters as the cornerstone of a meaningful cultural-based research. This model was first experimented in 2019 between the University of Luxembourg and the East China Normal University in Shanghai. It is being implemented as an international mobility project between the University of Oslo, the East China Normal University and the Federal University of Bahia by the authors of the present article. The idea is very simple: pairs of students from different cultural and academic backgrounds were created to work together on a joint small empirical study for their MA theses (Xu & Marsico, 2020). The research-tandem consists of paring one host student working as an insider or “local informant” with a guest student from a different country as outsider to make observations in the hosting context. The complementary cultural understanding helps, on the one hand, the “outsider” who is guided into the culturally situated comprehension of local meanings avoiding an ethnocentric or superficial gaze. On the other hand, the “insider” may be forced to dissect the take-for-grant set of meanings in their own culture. The educational device crosses the borders that the more traditional cross-cultural studies take for granted. If the research-tandems are established between students of the Global North and the Global South, for instance, they can help to deconstruct the single direction that mostly characterizes cross-cultural studies, in which the comparison is made taking the Global North’s perspective. Seldom one could find a cross cultural study between, for instance, USA and Brazil, in which the dominant perspective is Brazilian. The first experiences of research-tandem students have been documented in the book edited by Xu & Marsico (2020).

The research-tandem experience originates from the ideal of a borderless university, a scientific endeavor highly sensitive to the local meanings human beings produce in their everyday mundane activities and highly resistant to the idea of disciplinary and national (istic) boundaries in the creation of knowledge. This ideal has been cultivated through an international network of excellence on innovative learning, teaching environments and practices called “IBEF- Ideas for the Basic Education of the Future”. IBEF is the concretization of a utopia and is a milestone of the international programme promoted within the framework of cultural psychology of education (Marsico, 2017). IBEF is ideally located at East China Normal University that coordinates a large network of Universities (Aalborg University, DK; Oslo University, Norway, University of Salerno, Italy; Federal University of Bahia, Brazil and Luxembourg University among others). The IBEF network of excellence aims at looking beyond the current trends in basic education and at identifying the most innovative and edge ideas, to study and understand how to implement them on the long term.

The IBEF philosophy that produced an idea such as the research-tandem is the answer to Szulevics, Lund and Lund (2021) concerns. It is not the choice of the method or the choice of the theory that limits the novelty and depth of students’ understanding of human psyche. The qualitative/quantitative controversy is pointless if one considers the goal of psychology is to understand human meaning-making (Valsiner, 2021). Human experience is a matter of quality. Thus, quantitative methods are a sub-category of qualitative methods. The former use numbers to represent human meaning while the latter use words and image. The illusion of digital *versus* analogic creates the sense of “objectivity” that quantitative methods seem to have. However, they are complementary ways to represent the complexity of human *psyche* which is never reducible to simple variables. If Szulevics, Lund and Lund (2021) distinguishes between two psychologies only on the basis of the quality/quantity binary, they miss the point. The issue of psychology in general is its capability of understand the complexity of meaning-making without forcing it into a monological interpretive framework, namely the one promoted by the Global North. The combined effect of a pseudo-empirical compulsive accumulation of data – no matter if qualitative or quantitative – with the current reproductive neoliberal model of higher education are way more dangerous (Nussbaum, 2010). Psychology students, in Denmark as in many other countries, are socialized to become technicians and to conform to normative models, rather than becoming humanists able to produce innovative ideas with scientific rigor and to dare challenging the state of things. A vision of a future-oriented higher education should support the development of students’ full potential beyond mere fulfilling of social and market expectations. The philosophy on which IBEF was built regards the open nature of cultural dialogue. Knowledge does not follow the second law of thermodynamics. Indeed, the more it expands, the more it becomes dense; and the more it is shared the more it grows. We can concretely imagine a higher education in which students are free to roam between wisdoms and places. A universal system of credits should be meant not to produce interchangeable and standardized students-as-products that can be placed somewhere in the labour market. It should be understood as a way to support the freedom of students to move worldwide to develop new knowledge and follow their curiosity. A university without borders would go across national and disciplinary boundaries, making the current neoliberal organization outdated.

## Data Availability

Data sharing is not applicable to this article as no new data were created or analysed in this study.
